# Community Clozapine Initiation Practice

**DOI:** 10.1192/bjo.2024.648

**Published:** 2024-08-01

**Authors:** Kabir Yisa, Adel Ghaly, Louise Cumming, Taiwo Adenusi

**Affiliations:** Lancashire and South Cumbria NHS Trust, Preston, United Kingdom

## Abstract

**Aims:**

To establish the proportion of CMHT Preston service users with schizophrenia who met the NICE standard (CG 178) of being offered clozapine after inadequate response to treatment with at least two antipsychotic drugs.

**Methods:**

Inclusions – Service users on the CMHT Preston caseload with schizophrenia who attended outpatient clinic between January and June 2023.

Exclusions – Organic psychosis and non-schizophrenic/unspecified psychosis.

Sample size – 50.

Sampling – First 50 service users with established diagnosis of schizophrenia.

Data collection – Retrospective case-note audit from electronic patient records.

Data analysis – Quantitative.

**Results:**

45 service users (90%) met the clozapine eligibility criteria of not responding adequately to or tolerating at least 2 other antipsychotic medications while 5 service users (10%), did not meet the criteria. The proportion of eligible service users who were offered clozapine, and therefore met the standard, was approximately 64%, representing 29 out of the 45 eligible service users. Approximately 36%, representing 16 eligible service users, were not offered clozapine. In one isolated case, a service user who had only 1 previous antipsychotic trial and therefore did not meet the eligibility criteria, was offered clozapine. No reason was given in 13 out of the 16 service users who were not offered clozapine despite meeting the eligibility criteria. In the remaining 3 service users in this group, 2 were not offered clozapine because of cardiac problems and 1 was not offered because of significant history of poor compliance with antipsychotic medications. Furthermore, 25 eligible service users (86%) of those who were offered clozapine went on to initiate it with only 4 service users (14%) in this group not going ahead to initiate clozapine. In all 4 service users who did not initiate clozapine after being offered, the reason given was that the service users declined it.

**Conclusion:**

The findings from this audit indicate that a considerable proportion (64%) of CMHT Preston service users with schizophrenia are being offered clozapine in line with the NICE standard, and 86% of those offered went on to initiate clozapine. However, there is room for improvement in terms of offering and ultimately initiating clozapine in a timely manner as evident from the findings which highlighted an average of three antipsychotic trials before eligible service users were offered clozapine. The existing established local clozapine community initiation pathway can potentially be optimised to improve clozapine access and ultimately enhance clinical outcomes for this subset of service users.

